# Unusual Presentations of Pseudophakic Pupillary Block: A Report of Two Cases

**DOI:** 10.7759/cureus.89875

**Published:** 2025-08-12

**Authors:** Yie Ping Chin, Zhen Ning Low, Nurul Ashikin Abdullah, Nor Azita Ahmad Tarmizi, Roslinah Muji

**Affiliations:** 1 Ophthalmology, Hospital Kuala Lumpur, Kuala Lumpur, MYS; 2 Ophthalmology, Hospital Selayang, Kuala Lumpur, MYS

**Keywords:** angle closure, glaucoma, pseudophakic, soemmering’s ring, ultrasound biomicroscopy

## Abstract

Pseudophakic angle closure is an uncommon complication following cataract surgery. We report two cases with distinct underlying mechanisms, diagnosed through careful anterior segment evaluation and ultrasound biomicroscopy (UBM). The first case involved a patient with primary angle closure suspect (PACS) who developed a pseudophakic pupillary block due to a post-inflammatory membrane following treatment for aqueous misdirection. The second case highlighted a pupillary block induced by a Soemmerring’s ring. These cases underscore the importance of early UBM use in pseudophakic eyes presenting with angle closure, as it facilitates prompt identification of the etiology and anatomical changes, guiding appropriate clinical management.

## Introduction

Secondary angle closure in pseudophakic eyes is rare. Various etiologies, including aqueous misdirection, Soemmerring’s ring, and inflammatory membranes, can contribute to its development. The pressure rise may be transient or permanent, depending on the underlying cause. Pupillary block remains the most common cause of angle closure following cataract surgery and intraocular lens (IOL) implantation [1,2]. This condition arises when the pupillary aperture becomes obstructed by any surface it contacts, either anterior or posterior [1]. Potential causes include malignant glaucoma, Soemmerring’s ring, iris-IOL contact, pupillary block from air, silicone oil, vitreous, or iris-postoperative membrane adhesion [1].

Here, we present two cases demonstrating distinct mechanisms of pseudophakic pupillary block. The first case involves a patient with primary angle closure suspect (PACS) who developed a post-inflammatory membrane shortly after treatment for aqueous misdirection. Although peripheral iridotomy (PI) was visible on transillumination, its functional patency was compromised. In the absence of UBM at presentation, the exact mechanism could not be confirmed; however, the recurrence may have been due to cyclitic membrane formation resulting in seclusio pupillae. The second case illustrates pupillary block induced by a Soemmerring’s ring.

These cases highlight the value of early UBM to determine the etiology of secondary angle closure and the need to consider occlusive membranes or inflammatory changes even when the PI appears patent. Soemmerring’s ring formation, although uncommon, should remain in the differential diagnosis for pseudophakic eyes with angle closure.

## Case presentation

Case 1

**Figure 1:**
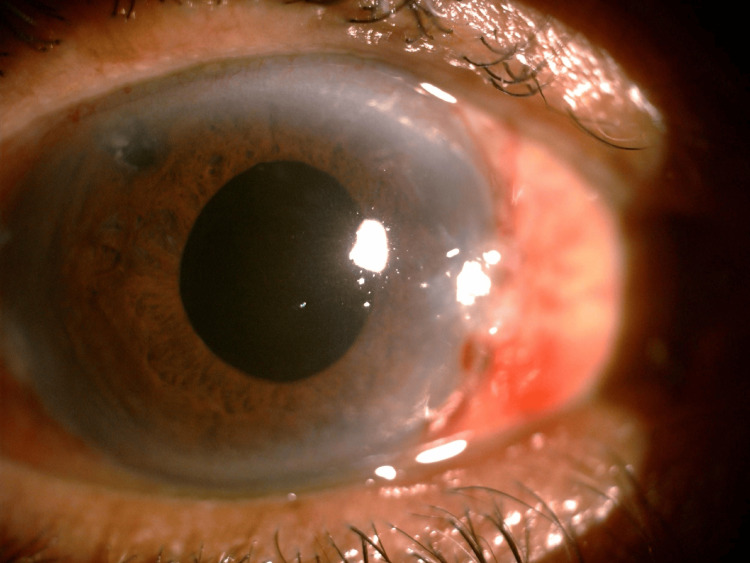
Slit-lamp photograph of the right eye at initial presentation showing a very shallow anterior chamber and a visible peripheral iridotomy at the 10 o’clock position

A 74-year-old man with right eye (RE) PACS underwent uneventful cataract surgery five years prior and a laser peripheral iridotomy (PI) two years ago. At routine follow-up, slit-lamp examination revealed a shallow anterior chamber (AC) (Figure 1) with elevated intraocular pressure (IOP) of 28 mmHg. Gonioscopy showed Shaffer grade 0 in all quadrants. The PI appeared patent on transillumination, and B-scan ultrasonography showed no posterior segment abnormalities.

A presumptive diagnosis of aqueous misdirection was made. Neodymium-doped yttrium aluminum garnet (Nd:YAG) anterior hyaloidotomy was performed, and topical antiglaucoma agents, along with atropine sulphate 1%, were initiated. Despite treatment, the IOP increased further to 46 mmHg.

The patient subsequently underwent pars plana vitrectomy, surgical capsulotomy, and PI enlargement. Intraoperatively, the PI was found to be obstructed by residual lens capsule and cortical remnants. The AC deepened immediately postoperatively, and IOP normalized. However, two weeks later, the patient experienced a recurrence of angle closure with an IOP of 36 mmHg. A thin fibrin membrane occluding the PI was observed. This was treated with Nd:YAG laser membranectomy and PI re-enlargement. Since then, the IOP has remained stable at 10 mmHg with monotherapy.

Case 2

A 16-year-old Malay boy was diagnosed with bilateral idiopathic intermediate uveitis (IU) and uveitic cataracts at age 10. He underwent bilateral surgical synechiolysis, lens aspiration, and posterior chamber IOL implantation under general anesthesia. Extensive investigations excluded infectious or systemic inflammatory etiologies. He responded well to tapering systemic corticosteroids.

**Figure 2:**
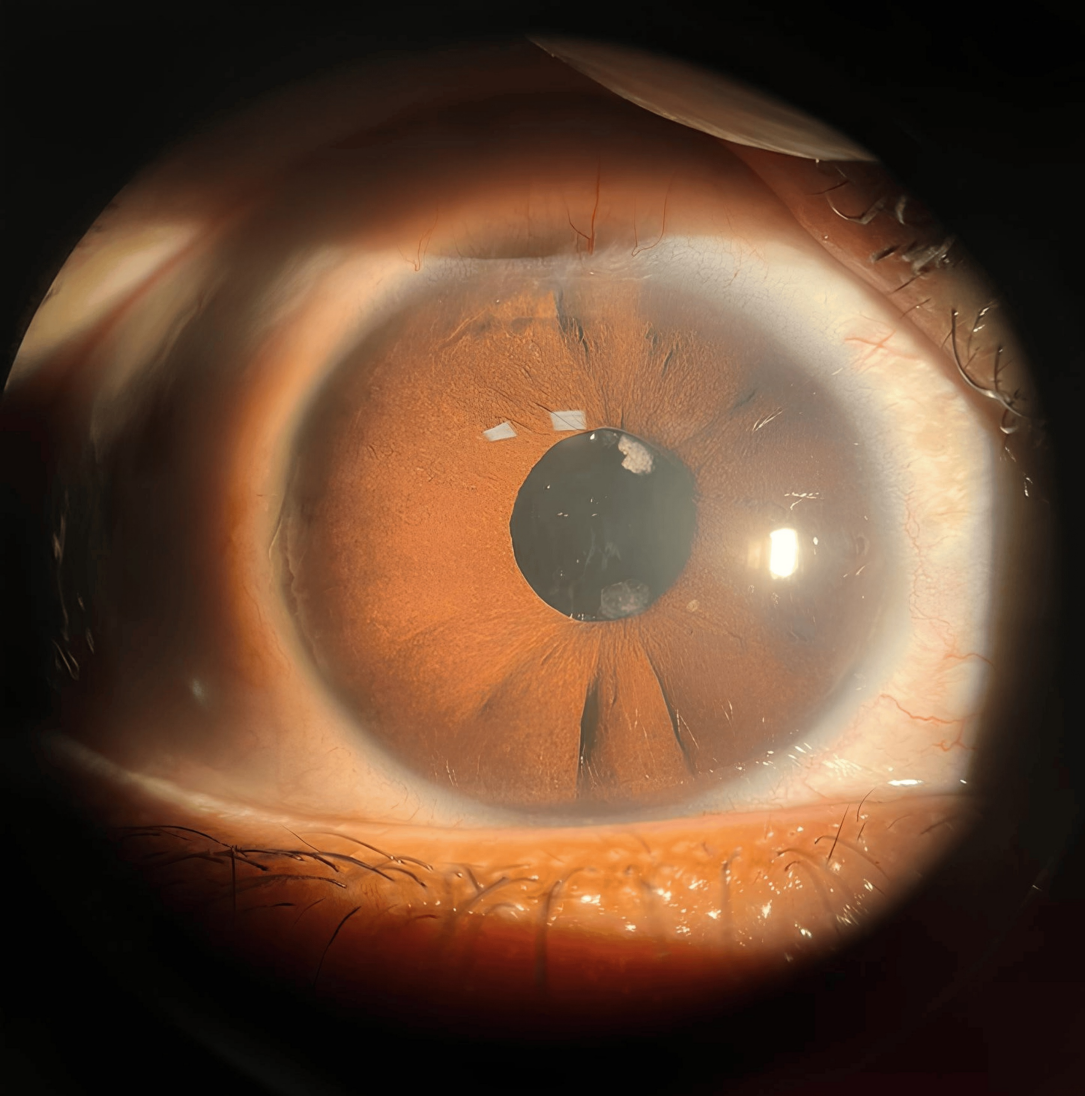
Anterior segment photo showing residual lens matter at 1 and 6 o’clock with iris bowing temporally

**Figure 3:**
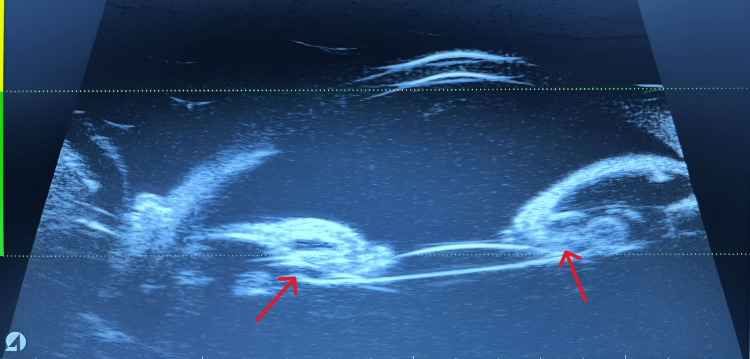
UBM showing the presence of an ovoid hyperechoic lesion as indicated by the red arrow (Soemmerring’s ring), which obliterated the sulcus space and led to the formation of iris bombe UBM: ultrasound biomicroscopy

At five years postoperatively, his right eye IOP rose to 32 mmHg during routine follow-up. Examination revealed residual lens matter and anterior bowing of the iris (Figure 2), suggestive of a Soemmerring’s ring causing secondary angle closure glaucoma. UBM confirmed the diagnosis (Figure 3). The Soemmerring’s ring was surgically removed. Postoperatively, IOP normalized, antiglaucoma drops were discontinued, and best-corrected visual acuity improved to 6/12 in the right eye and 6/9 in the left eye.

## Discussion

In Case 1, the cause of angle closure attack was initially postulated due to aqueous misdirection based on the elevated IOP, diffuse shallowing of the anterior chamber, a patent PI, a normal posterior segment, and a prior history of cataract surgery.

The pathophysiology of aqueous misdirection remains poorly understood. It is primarily attributed to an imbalance between transvitreal pressure and aqueous humor outflow, resulting in an anatomical disruption of the ciliary body, anterior hyaloid face, and vitreous. This disruption leads to a forward shift of the iris-lens diaphragm, causing secondary angle closure [3]. This condition can occur following various ocular surgeries, including filtration surgery, phacoemulsification [4], and laser procedures such as PI [5].

Treatment options for aqueous misdirection include medical therapy, laser procedures such as Nd:YAG laser capsulotomy and anterior hyaloidectomy, and surgical interventions. Vitrectomy may be necessary in cases of uncontrolled IOP or progressive anterior chamber shallowing [6], as observed in this patient.

Intraoperative findings revealed that the PI was obstructed by the lens capsule and retained cortical matter. In this case, the blockage occurred when an inflammatory membrane occluded the PI, explaining why the IOP did not reduce despite treatment. During slit-lamp examination, we had determined that the PI was patent using the transillumination technique. However, this case highlights that a positive transillumination test does not guarantee true patency, as residual, sparse pigmented stroma may remain even after the pigmented epithelium has disappeared [6].

In this case, the patient developed another episode of pseudophakic pupillary block post-vitrectomy due to the presence of fibrin membrane covering the PI opening, which may result from postoperative ocular inflammatory reactions [7].

UBM could only be performed later, after the vitrectomy and enlargement of the PI, due to unavailability at the center. Early use of UBM in this case could have distinguished between true aqueous misdirection and mechanical pupillary block by directly visualizing anterior hyaloid configuration, capsular remnants, or inflammatory membranes.

In case 2, the angle closure attack was due to the formation of a Soemmerring’s ring, which can typically occur after cataract surgery with or without IOL implantation. This happens when the central region of the anterior lens capsule is exposed, leading to the deposition and proliferation of the remaining lenticular epithelial cells in the peripheral part of the capsular bag. This process results in the formation of a thick circumferential structure at the level of the lens [8]. The resulting mass effect can push the iris forward, precipitating pupillary block, particularly in younger patients with more pronounced proliferative activity.

In this case, early use of UBM was crucial in confirming the diagnosis by demonstrating the anatomical relationship between the angle, IOL, Soemmerring’s ring, and ciliary complex. Additionally, it provided visualization of the extent of the Soemmerring’s ring, which assists in determining the appropriate treatment strategy, as shown in Figure 3.

## Conclusions

Secondary angle closure in pseudophakic eyes is uncommon but may arise from mechanisms such as aqueous misdirection, retained lens material, inflammatory membranes, or Soemmerring’s ring. This report highlights the importance of comprehensive assessment, including UBM, to accurately identify the etiology and guide management. A patent PI on transillumination does not guarantee functionality. Soemmerring’s ring, although rare, should be included in the differential diagnosis in any pseudophakic patient presenting with angle closure.
